# Dynamics of deep-submarine volcanic eruptions

**DOI:** 10.1038/s41598-022-07351-9

**Published:** 2022-02-28

**Authors:** Eric L. Newland, Nicola Mingotti, Andrew W. Woods

**Affiliations:** Department of Earth Science, BP Institute, University of Cambridge, Cambridge, UK

**Keywords:** Solid Earth sciences, Volcanology

## Abstract

Deposits from explosive submarine eruptions have been found in the deep sea, 1–4 km below the surface, with both flow and fall deposits extending several km’s over the seafloor. A model of a turbulent fountain suggests that after rising 10–20 m above the vent, the erupting particle-laden mixture entrains and mixes with sufficient seawater that it becomes denser than seawater. The momentum of the resulting negatively buoyant fountain is only sufficient to carry the material 50–200 m above the seafloor and much of the solid material then collapses to the seafloor; this will not produce the far-reaching fall deposits observed on the seabed. However, new laboratory experiments show that particle sedimentation at the top of the fountain enables some of the hot, buoyant water in the fountain to separate from the collapsing flow and continue rising as a buoyant plume until it forms a radially spreading intrusion higher in the water column. With eruption rates of 10$^6$–10$^7$ $\hbox {kg s}^{-1}$, we estimate that this warm water may rise a few 100’s m above the fountain. Some of the finer grained pyroclasts can be carried upwards by this flow and as they spread out in the radial intrusion, they gradually sediment to form a fall deposit which may extend 1000’s m from the source. Meanwhile, material collapsing from the dense fountain forms aqueous pyroclastic flows which may also spread 1000’s m from the vent forming a flow deposit on the seabed. Quantification of the controls on the concurrent fall and flow deposits, and comparison with field observations, including from the 2012 eruption of Havre Volcano in the South Pacific, open the way to new understanding of submarine eruptions.

## Introduction

Explosive eruptions are known to have occurred in many submarine environments over geological history. The associated deposits suggest that the eruption of fragmented mixtures of magma and seawater form ascending flows which eventually feed submarine flow and fall deposits^1–16^, while less fragmented eruptions of larger pumice clasts can lead to pumice rafts spreading out on the sea surface^17–21^.

The dynamics of eruptions on the seabed and the associated fragmentation processes are poorly understood, but there is evidence of highly fragmented magma issuing from deep-submarine eruptions and that these lead to fine-grained deposits distributed over 100–1000’s m on the seafloor: important examples include the deposits found on the Gakkel ridge in the Arctic^12,22,23^; the Marsili Seamount, Italy^5,6^; the Gorda and Juan de Fuca Ridges^9,11,24^; Loihi, Hawaii^10,25,26^; and the well documented ash deposit, specifically Subunit 3 of the Ash with Lapilli unit, produced during a later explosive stage of the 2012 eruption of Havre volcano, in the South Pacific north of New Zealand^14–16^.

The eruption and mixing of hot magma directly into seawater may initially produce a very hot and buoyant mixture relative to the surrounding ambient fluid since the density of very hot water or water vapour is small, even at depths of a few km. However, upon continual mixing with seawater, the temperature falls and owing to the non-linear dependence of the water density on temperature^27^ the mixture can eventually become denser than the ambient water^4^. Using a model for turbulent entrainment into a jet^28,29^ we present new calculations which suggests this occurs within a few 10’s m above the source. The subsequent motion is essentially that of a turbulent, particle-laden fountain, whose bulk density exceeds that of the surrounding seawater, but in which the water is hot and hence of lower density^1–4,30–32^. The ascent of this negatively buoyant fountain is gradually arrested by gravity and the majority of the erupting material is likely to collapse back to the seafloor. This raises the fundamental question as to how distal fall deposits can develop from such eruptions. Here we present a series of new laboratory experiments to elucidate the dynamics of these complex multi-phase flows, illustrating that some of the hot and buoyant water can rise from the top of this collapsing fountain, carrying some particles upwards through the overlying water column. Owing to the ambient stratification, this flow eventually reaches a maximum height and intrudes radially into the ambient fluid, leading to a radial fall deposit. We compare this picture of the flow dynamics with field observations reported in the literature, including the recent ash deposits from the Havre eruption in 2012.

For context, we note that Barreyre et al.^2^ proposed a model for the dispersal of pyroclastic material from such eruptions based on the idealised single-phase model of a turbulent buoyant plume, as originally applied to describe a hydrothermal plume^33^. Such models for the ascent of a positively buoyant plume and the subsequent dispersal of pyroclasts from a radially spreading neutral cloud, have been applied to specific eruptions such as Havre, 2012^15^. Also classical plume theory has been used to estimate the heat flux produced during submarine eruptions^34^. In that latter study the authors proposed that hot hydrothermal water released from the seafloor during an eruption, coupled with heat transfer from lava spreading on the seafloor produced a plume which carried a small fraction of the pyroclasts upwards through the water column.

However, these earlier models did not address the details of the density evolution of a mixture of hot dense pyroclasts and seawater. Here we show that following the eruption of pyroclasts from the seafloor, the mixing with seawater eventually leads to the formation of a dense fountain and this forms the topic of the present work.

## Results

### Source conditions

Assuming good thermal contact, then as a mass flux of water $Q_w$ mixes with the mass flux $Q_m$ of erupting fragmented magma, the mixture temperature *T* is given by heat conservation

1 $$\begin{aligned} {[}L + C_{pm} (T_0 - T ) ] Q_m = h_w(T,P) Q_w \end{aligned}$$

where *L* is the latent heat of the magma, with value of order 3 $\times$ $10^5$ $\hbox {Jkg}^{-1}$^35^, and $C_{pm}$ is the specific heat of magma and $h_w(T,P)$ is the enthalpy of the water or water vapour which depends on the temperature and pressure. $C_{pm}$ has an approximate value of $1000\, \hbox {J kg}^{-1}\hbox {K}^{-1}$^35^ and $T_0$ is the temperature of the erupted magma with an assumed value of $1400\, {}^{\circ }\hbox {C}$. We also assume that the mixture cools below the solidification temperature of the molten magma and therefore the density of the water-magma mixture is given by (c.f.^36^)

2 $$\begin{aligned} \rho = (Q_m + Q_w)\left( \frac{Q_m}{\rho _m}+\frac{Q_w}{\rho _w}\right) ^{-1} \end{aligned}$$

To estimate the density of hot water as a function of the pressure and temperature, we follow the data in Rogers and Mayhew^37^. For pressures below that of the critical point, there is a change in phase from vapour to liquid as the temperature falls below the Clausius Clapeyron curve and the density increases significantly, while for pressures above the critical point, the density smoothly evolves from very small values at high temperature towards much larger values as the temperature approaches that of seawater. For simplicity, we neglect the effect of salt, which is present in seawater, on the thermodynamic properties at high temperatures, close to or above the saturation point^38^.

**Figure 1:**
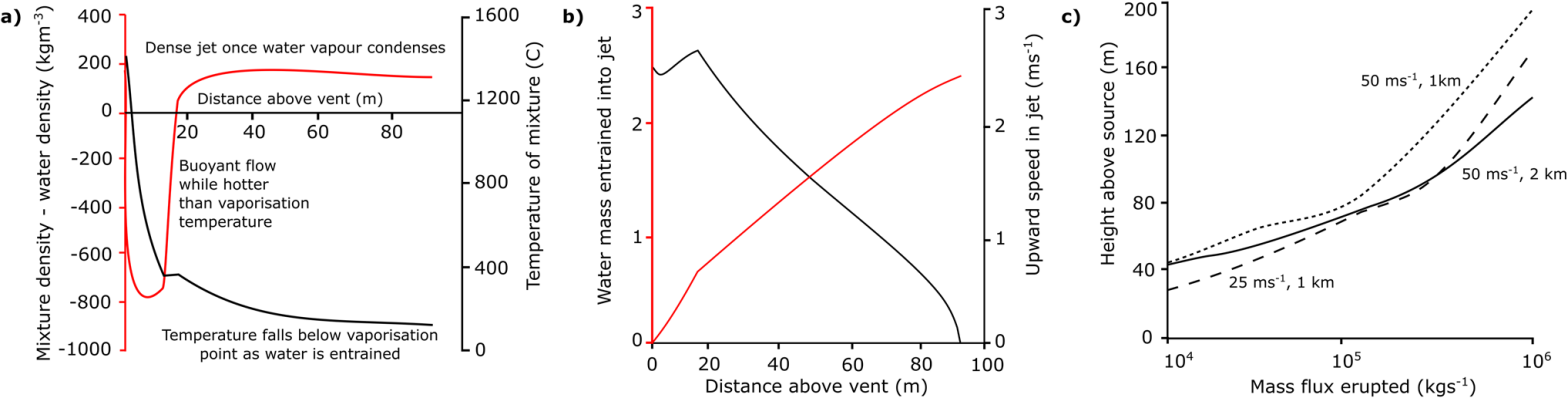
(**a**) Density of the jet relative to the ambient water (red line) and temperature of water-solid mixture (black solid line) calculated from the jet model described in the text; (**b**) entrained mass flux of water as a fraction of the mass eruption rate, (red line) and upward speed of the fountain (black line) as a function of distance above the vent, calculated from the jet model, for an eruption rate of $10^6\,\hbox {kg s}^{-1}$, and eruption temperature of 1400 °C, from a vent at a depth of 2 km below the surface. (**c**) Maximum height of rise of a submarine fountain as a function of the erupted mass flux, for eruption speeds for 25 and $50\, \hbox {ms}^{-1}$ at a depth of 1 km below the sea surface, and for $50 \,\hbox {ms}^{-1}$ at 2 km below the sea surface.

At depths of 1000 m or more below the sea surface, both $\hbox {CO}_2$ and $\hbox {H}_2\hbox {O}$ may be exsolved from the magma but, with pressures of 10’s MPa or more, volatiles have a relatively small impact on the buoyancy of the water-magma mixture compared to the presence of the particles. Indeed, in the submarine deposits observed at Gakkel ridge^12^, the vesicularity was less than about 5$\%$ and such clasts are dense relative to the surrounding water. In contrast, in shallow submarine eruptions, pumices may be more vesicular; with sufficiently large vesicularity they may be buoyant leading to different dynamics^17–20^. Here we focus on the common case in which even the vesicular pyroclastic material is denser than the seawater.

We have coupled the above model of the heat transfer with a model for a buoyant jet, as given by the classical relations for the mass flux, $Q(z) = \pi q(z)$, and momentum flux, $M(z) = \pi m(z)$,

3 $$\begin{aligned} \frac{d q}{dz} = 2 \alpha m^{1/2} \hbox { and } \frac{d m}{dz} = g(\rho _a - \rho ) b^2 \end{aligned}$$

where $\rho _a$ is the density of the ambient seawater, $q = \rho ub^2$, $m = \rho u^2 b^2$, *u* and $\rho$ are the horizontally averaged speed and bulk density of the jet of radius, *b*. The bulk density depends on the mass fraction of pyroclasts, $\phi_p$, of density $\rho _p$ and fluid, of density $\rho _w$ according to

4 $$\begin{aligned} \rho = \left[ \frac{\phi_p }{\rho _p} + \frac{1 -\phi_p }{\rho _w}\right] ^{-1} \end{aligned}$$

Also, $\alpha$ is the entrainment coefficient which for simplicity we take to have value 0.1 in the present model; in fact, the detailed value of $\alpha$ does vary between jets and plumes^28^, and may be influenced by non-boussinesq effects owing to the large density differences in this system, but we do not expect such variations to change the leading order processes described herein. It would be interesting to refine this component of the model with further detailed experiments^39–43^.

We have solved the above equations for the motion of the jet numerically. Typical model results are shown in Fig. 1, where we see the evolution of the temperature, speed, buoyancy and water mass fraction of the fountain as a function of distance from the source for a typical example calculation of an eruption of $10^6$ $\hbox {kg s}^{-1}$ at a depth of 1 km below the sea surface (Fig. 1a,b). As water is mixed into the fountain, the water is heated to very high temperatures and expands to very low density leading to the bulk flow becoming buoyant relative to the seawater. However, after about 20 m from the vent, a sufficient mass of water has been entrained that the mixture is now cooler and the water density has increased, so that the bulk density becomes greater than that of the surrounding water. The flow then evolves as a negatively buoyant fountain, and in this example, the fountain speed falls to zero as it reaches a maximum height of about 100 m above the seafloor. This maximum height is sensitive to the initial speed of the flow and the depth of the water, owing to the different pressure and hence differences in the detailed thermodynamic properties of the water. In Fig. 1c, we illustrate the variation in the maximum height of rise between a 1 and 2 km deep volcano, and between eruptions with source velocities of 10, 25 and $50\,\hbox {ms}^{-1}$. Such differences in source velocity are likely the result of differences in the volatile content of the magma (cf.^4^).

In the deep sea, the density stratification is relatively weak, and so the fountain ascent is largely limited by the negative buoyancy; in the model we do account for the decrease of the ambient density with height, according to the simplified model that the Brunt-Väisälä frequency has value $N=0.001$ $\hbox {s}^{-1}$ typical of the deep sea (e.g.^33^) where

5 $$\begin{aligned} N^2 = -\frac{g}{\rho _a} \frac{d \rho _a}{dz} \end{aligned}$$

Note that with very strong eruptions, the stratification would begin to have an impact on the height of rise^32,40,44^. We also note that the speed of rise of the fountain is typically in excess of 1 $\hbox {ms}^{-1}$ except in the upper few metres prior to the flow coming to rest. Given that the fall speed of the pyroclasts in submarine eruptions have been recorded in the range is 0.01–$0.1\, \hbox {ms}^{-1}$^2,12,14,15^, we therefore expect that the pyroclasts will be carried up to the top of the fountain (cf.^45^). However, the subsequent dynamics of the pyroclast-laden flow are complex since, although the bulk density of the fountain is larger than the ambient fluid, the fluid phase in the fountain is buoyant. We now present a series of new laboratory experiments to explore the dynamics of such flows.

**Figure 2:**
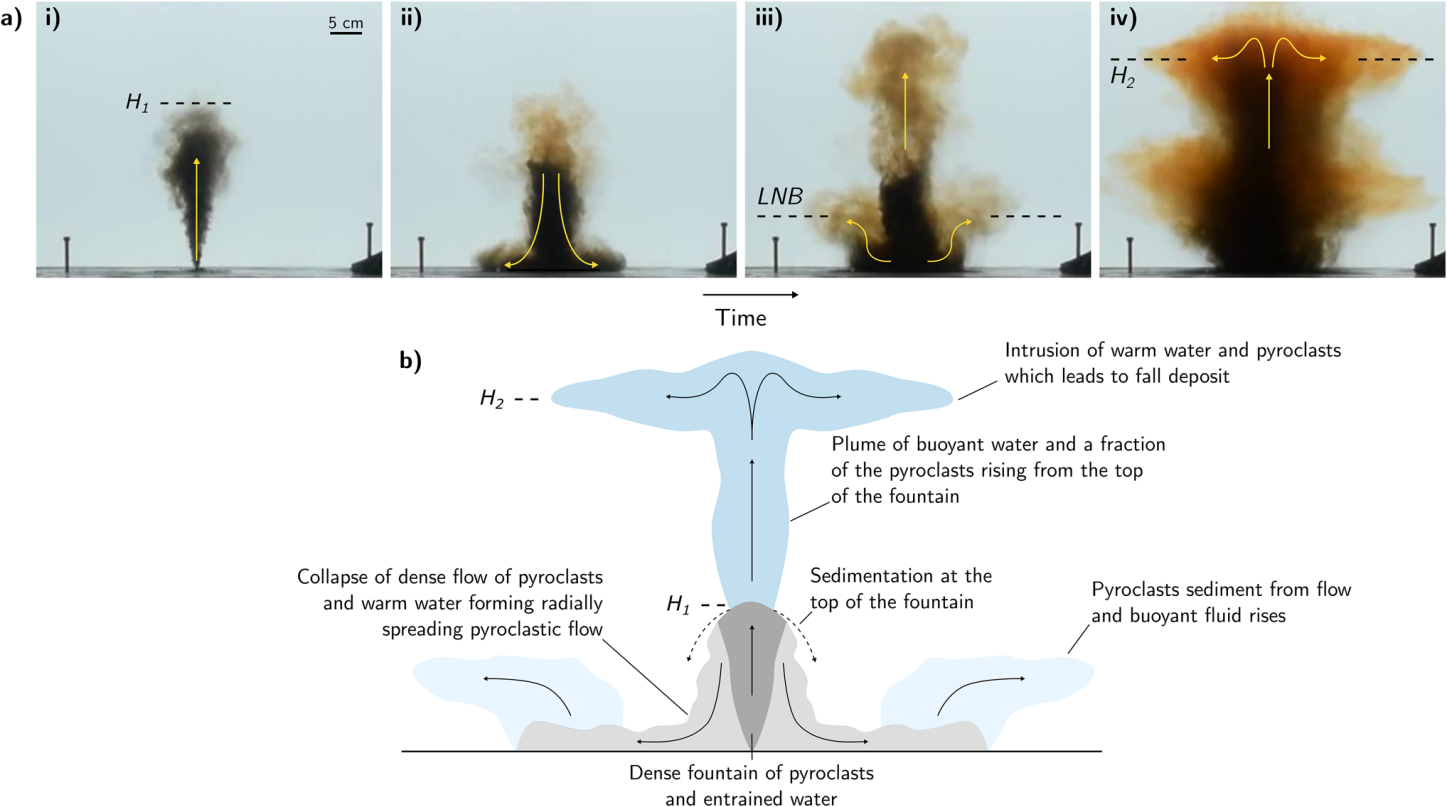
(**a**) Experimental images displaying the dynamics of a particle-laden fountain with positively buoyant interstitial fluid in a weak ambient stratification. The frames (i–iv) were taken at times 5 s, 10 s, 15 s and 60 s after the initial injection. Images show both the collapse of the particle-laden fountain to form a flow deposit and the rise of buoyant fluid and particles from the top of the fountain. Panel (iii) shows the formation of a lower intrusion (*LNB*) as fluid rises from the gravity current and panel (iv) shows the formation of an upper intrusion $H_2$ as a result of particle-fluid separation at the top of the fountain. (**b**) Schematic cartoon illustrating the primary dynamics of a deep-submarine explosive eruption.

**Table 1:** Experimental parameters for particle fountains.

Exp.	$M_0$ ($\times 10^{-6}$)	*Re*	*Fr*	$\rho _f$	$\rho _a$	$C_0$	$D_p$ ($\times 10^{-6}$)	$v_s$ ($\times 10^{-3}$)	*U*	*N*	$\phi$
1	3.14	2000	27.8	1000	1044	0.031	63.0	5.40	0.18	0.32	–
2	7.85	2020	17.8	1010	1030	0.019	22.8	0.69	0.02	–	–
3	7.85	2020	17.8	1010	1030	0.019	53.0	3.75	0.13	–	–
4	7.85	2020	17.8	1010	1030	0.019	75.0	7.51	0.25	–	–
5	7.85	2020	17.8	1010	1030	0.019	106	15.0	0.50	–	–
6	7.85	2020	17.8	1010	1030	0.019	150	30.0	1.01	–	–
7	7.85	2020	17.8	1010	1030	0.019	212	60.0	2.01	–	–
8	1.27	1590	10.2	1000	1000	0.007	75.0	7.51	0.27	–	–
9	1.27	1590	10.8	1000	1001	0.007	75.0	7.51	0.28	–	–
10	1.27	1590	11.5	1000	1003	0.007	75.0	7.51	0.31	–	–
11	1.27	1590	12.3	1000	1004	0.007	75.0	7.51	0.33	–	–
12	1.27	1590	13.3	1000	1006	0.007	75.0	7.51	0.35	–	–
13	1.27	1590	14.5	1000	1007	0.007	75.0	7.51	0.39	–	–
14	1.27	1590	16.3	1000	1009	0.007	75.0	7.51	0.43	–	–
15	1.27	1590	18.8	1000	1010	0.007	75.0	7.51	0.50	–	–
16	1.27	1590	23.1	1000	1011	0.007	75.0	7.51	0.61	–	–
17	1.27	1590	32.6	1000	1013	0.007	75.0	7.51	0.87	–	–
18	2.82	1914	16.9	1010	1030	0.019	22.8	0.69	0.02	–	0.00
19	2.82	1914	16.9	1010	1030	0.019	22.8–212	0.69–60	0.02–2	–	0.25
20	2.82	1914	16.9	1010	1030	0.019	22.8–212	0.69–60	0.02–2	–	0.50
21	2.82	1914	16.9	1010	1030	0.019	22.8–212	0.69–60	0.02–2	–	0.75
22	2.82	1914	16.9	1010	1030	0.019	212	60	2.01	–	1.00

$M_0$ ($\hbox {m}^4\hbox {s}^{-2}$) is the source momentum flux, *Re* is the source Reynolds number, *Fr* is the source Froude Number, $\rho _f$ is the density of the fountain fluid ($\hbox {kg m}^{-3}$), $\rho _a$ is the density of the ambient fluid at the base of the tank ($\hbox {kg m}^{-3}$), $C_0$ is the initial concentration of particles in the fountain mixture, $D_p$ (m) is the particle diameter, $v_s$ ($\hbox {ms}^{-1}$) is the particle sedimentation speed, *U* is the dimensionless particle fall speed, *N* ($\hbox {s}^{-1}$) is the Brunt-Väisälä buoyancy frequency and $\phi$ is the fraction of particles with $U > 1$.

### Fate of warm fluid in fountain

The fluid in the fountain is heated by the pyroclasts, and hence becomes less dense than the ambient water. If some particles separate from the fluid at the top of the fountain, the residual fluid may become buoyant and rise. To demonstrate this process, we have carried out a series of analogue experiments in which small-scale fountains composed of particles and fresh water were emitted upward from the base of a tank filled with saline aqueous solution (details of the experimental set-up and technique are given in the “Methods” section).

Figure 2a, i–iv illustrates the typical evolution of an experiment (exp. 1, Table 1). First, a dense fountain rises from the source, and reaches a maximum height, $H_1$ (Fig. 2a, i). Here some of the fluid and particle mixture begins to fall back to the base of the tank (Fig. 2a, ii). On reaching the base of the tank, the mixture spreads out radially and particles sediment from the flow. The residual fluid in this current becomes buoyant and lifts off the base of the tank (Fig. 2a, iii). As the fluid lifts off, it becomes arrested by the ambient stratification and forms a low level intrusion at the level of neutral buoyancy, indicated *LNB* in Fig. 2a. Meanwhile, at the top of the fountain there is some sedimentation of particles from the fountain fluid, and this leads to the formation of buoyant fluid which rises from the top of the fountain, carrying the remaining particles higher into the water column (Fig. 2a, iii). Since the ambient fluid is weakly stratified, this fluid-particle mixture eventually reaches its level of neutral buoyancy, well above the top of the original fountain, and it intrudes at this new height, $H_2$ (Fig. 2a, iv). Particles then spread radially and gradually sediment through the water column. In summary, the particles in the fountain may follow two different pathways to the seafloor; either they form a flow deposit from the collapsed fountain flow as it spreads radially over the seafloor, or they form a fall deposit as they sediment from the upper intrusion $H_2$ which forms from the fluid which rises above the fountain (Fig. 2b).

**Figure 3:**
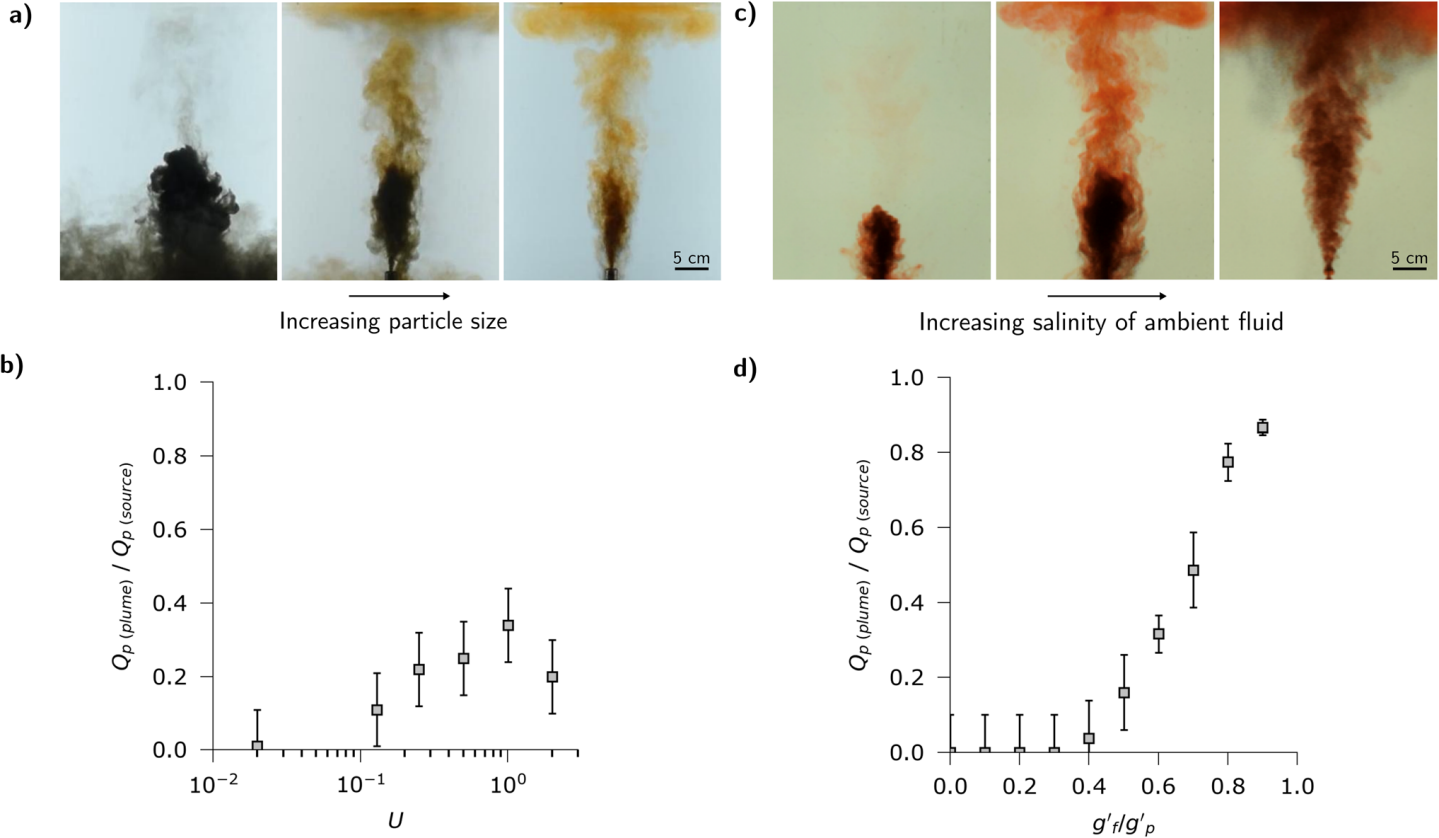
Experimental images illustrating how the fraction of fluid and particles rising from the top of the fountain varies with (**a**) the particle fall speed and (**c**) the density difference between the fountain fluid and the ambient fluid. All frames were taken 20 s after the initial injection. Experimental data showing the variation in the fraction of particles carried above the fountain as a function of (**b**) the dimensionless fall speed and (**d**) the buoyancy ratio of the interstitial fluid and particle load.

We now investigate the impacts of three key parameters which control the properties of the flow, namely (i) the particle size and settling speed, (ii) the density difference between the fountain fluid and the ambient fluid, and (iii) the effect of there being particles of different size and hence fall speed. We first present the results of a systematic series of experiments (exp. 2–7, Table 1) in which we used particles of one size and hence fall speed, $v_s$, but in which we varied this value from experiment to experiment, with all other properties fixed (Fig. 3a,b). These experiments show that the fraction of particles which are carried up from the fountain depend on the ratio of the fall speed of the particles to the characteristic speed in the fountain. For the idealised laboratory experiments, the fountain speed is given in terms of the buoyancy flux |*B*| and the momentum flux *M* according to the ratio $|B|^{1/2} M^{-1/4}$, and so the key ratio which determines whether particles are carried to the top of the fountain is

6 $$\begin{aligned} U = \frac{v_s}{|B|^{1/2} M^{-1/4}} \end{aligned}$$

When the particle fall speed is sufficiently large, $U>1$, we expect many particles to separate from the fountain fluid. As a result, a large fraction of the original fountain fluid rises above the fountain, although carrying relatively few particles as many have already sedimented. As the fall speed decreases, there is less separation of the particles from the fountain fluid and so more of the fountain fluid collapses to the ground. However, the fluid which is carried upwards has a larger particle load and so leads to a greater flux of particles being supplied to the upper intrusion. For very small fall speeds nearly all the particles collapse with the fountain fluid and so the flux of particles being carried upwards is again rather small.

A second control is associated with the density difference between the fountain fluid and the ambient fluid. Figure 3c,d presents the results of a second series of experiments (exp. 8–17, Table 1) in which the ratio of the positive buoyancy of the fluid, $g'_f = g (\rho _a-\rho _f)/\rho _a$, compared to the negative buoyancy of the particle load, $g'_p = g C_0(\rho _a-\rho _p)/\rho _a$ where $C_0$ is the initial particle concentration, in the fountain was systematically varied while the momentum flux and particle fall speed were fixed. If the fountain fluid is very buoyant then only a small fraction of particles need to sediment for the remaining mixture to become buoyant, whereas with a small density difference, the particle load in the fluid rising from the fountain is much smaller, and so the fall deposit is much smaller.

**Figure 4:**
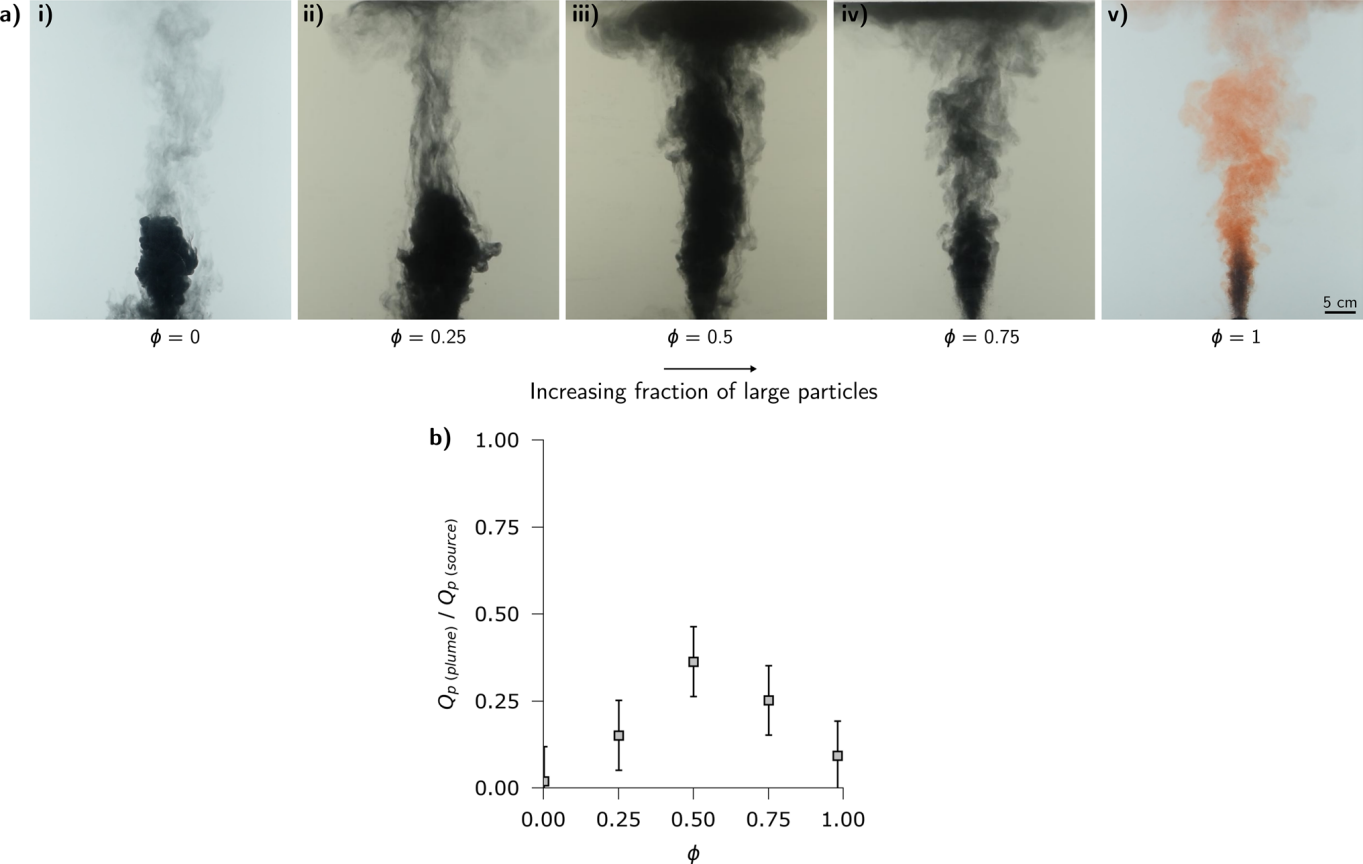
Experimental images (exp. 18–22) illustrating how the fraction of fluid and particles rising from the top of the fountain varies with the fraction, $\phi$, of large particles with $U>1$ in the mixture. All frames were taken at approximately 20 s after initial injection.

We also carried out a series of experiments in which we used particles of two different sizes and hence fall speeds, in order to determine the effect of there being a particle size distribution in the flow. In these experiments (exp. 18–22, Table 1) the particle-laden fountain contained a fraction $\phi$ of large particles ($U>1$) and a fraction $1-\phi$ of small particles ($U<1$), with $\phi$ taking values 0, 0.25, 0.5, 0.75 and 1. Figure 4a shows a series of experimental images for these 5 different values of $\phi$. We observe that as the fluid-particle mixture rises through the ambient fluid some of the large particles ($U>1$) separate from the flow and sediment to the tank floor, whereas the small particles ($U<1$) in the mixture remain coupled to the fountain fluid. With an intermediate value of $\phi$, the data suggest that a larger fraction of the solid material rises from the fountain. This may be understood in that with high values of $\phi$, most of the solid material is of larger size and so separates from the fountain, so that only a small fraction of the solid material rises in the fountain. In contrast, as $\phi$ approaches 0, following sedimentation of the larger particles, the mixture of fine particles and fountain fluid remains denser than the ambient, so that many of the small particles also collapse with the fountain. However, for intermediate values of $\phi$, once the larger particles have sedimented, the residual mixture of small particles and fluid is buoyant and so lifts off (Fig. 4b).

### Dispersal distances

The experiments have identified how particle size and particle load have a significant impact on the mass fraction of solids which may be carried upwards above the fountain in the plume of buoyant fluid. The subsequent dispersal of these particles depends on a balance between the sedimentation and the radial spreading of the plume fluid once it has been arrested by the ambient stratification (cf.^29^). In order to assess this balance, we first need to estimate the volume flux at the top of the plume rising off the fountain.

We can estimate the properties at the top of the fountain using the earlier model for the jet issuing from the volcano. This leads to a prediction of the temperature, volume flux of water and density of the mixture at the fountain top. For the parameter values typical of submarine eruptions considered herein (cf. Fig. 1), we find that at depths of 1–3 km if a fraction in excess of about 0.8–0.85 of the particles settle from the flow, then the remaining fluid typically becomes buoyant, and can rise up off the top of the fountain. To proceed, we assume that a fraction of the fountain fluid rises off the top of the fountain, carrying a fraction 0.1 of the particle load, and forms a buoyant plume. The motion of this plume is described by the earlier model equations (1–4) but using the properties at the top of the fountain as the source conditions.

We have conducted a parameter study to explore the sensitivity of the model predictions for the plume rise about the fountain, based on different eruption rates and different fractions of the fountain fluid rising off the fountain. These calculations show that when a fraction of 0.25–0.75 of the fountain fluid rises to form a plume, it can typically reach heights of 100–300 m above the top of the fountain with the height being limited by the ambient stratification. Also, the plume flow has a characteristic speed of a few metres per second which is an order of magnitude greater than the fall speed of typical pyroclasts found at Gakkel Ridge^2^ and also in the deposits from the Havre eruption of 2012^14,15^. In such a situation, we expect that pyroclasts can be carried to the top of the plume with the buoyant water.

On reaching the maximum height of rise, the fluid in the plume then spreads laterally into the ambient fluid, forming a radial intrusion. The volume flux *Q* supplying this intrusion is predicted by the plume model. As the flow spreads radially, the particles gradually sediment from the flow^46–48^. Veitch and Woods^49^ showed that owing to re-entrainment of particles into the plume, the concentration can increase, and that the typical length scale for sedimentation from the spreading flow is given by the balance of radial advection of particles and sedimentation, where the particle fall speed is denoted $v_s$, so that the particle concentration in the intrusion, *c*(*r*, *t*), varies as^29^

7 $$\begin{aligned} Q \frac{dc}{dr} = -2 \pi r v_s c \end{aligned}$$

leading to the balance

8 $$\begin{aligned} c(r) = c(r_o) \exp \left( - {\pi v_s r^2 / Q} \right) \end{aligned}$$

**Figure 5:**
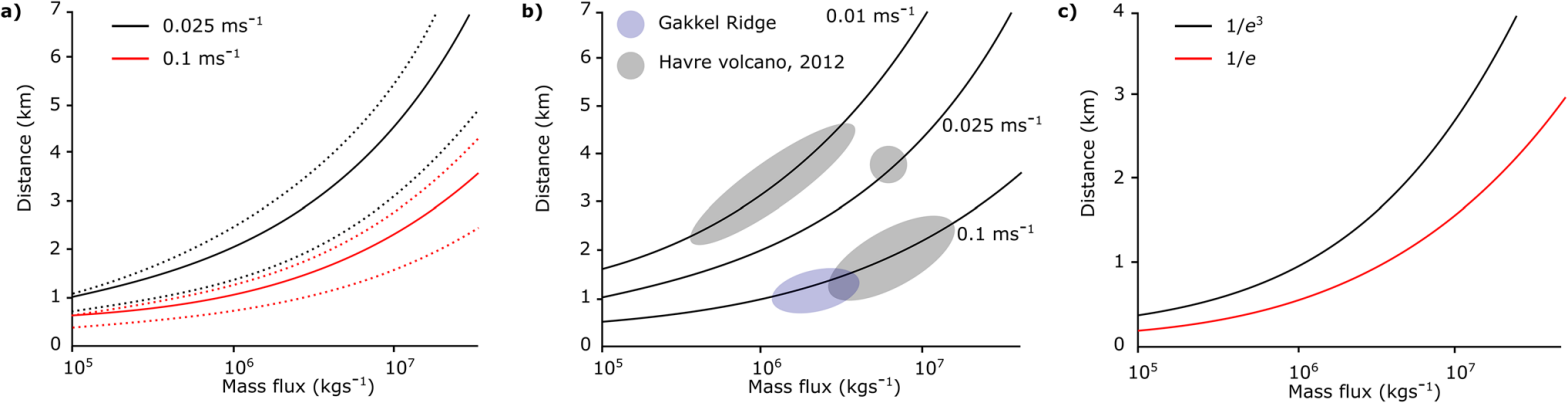
(**a**) Estimate of the range of particle dispersal distances of the fall deposit as a function of the mass eruption rate for particles with fall speed 0.025 and $0.1 \,\hbox {ms}^{-1}$. Curves are shown for the cases in which 0.75 (upper curve ), 0.5 (middle curve ) and 0.25 (lower curve) of the fluid in the fountain separates and rises up to form the plume; in each case it is assumed that a fraction 0.1 of the particle load in the fluid also rises up in the plume. (**b**) Estimate of the range of particle dispersal distances of the fall deposit as a function of the mass eruption rate, for particles of fall speed 0.1, 0.025 and $0.01\, \hbox {ms}^{-1}$, in the case that a fraction 0.5 of the fluid separates from the fountain. The circles illustrate the range of dispersal distances for sediment of particular fall speeds in Havre 2012 eruption deposit (grey circles) and the Gakkel ridge deposits (blue ellipse). (**c**) Estimate of the radial transport distance of particles in a radially spreading submarine flow. Curves correspond to the distance at which the particle load has decreased by a factor 1/*e* (red line) and $1/e^3$ (black line), for a particle with settling speed of $0.01 \,\hbox {ms}^{-1}$.

This relation identifies that 95 $\%$ of the particles have fallen out by a distance $r \approx (3 Q / \pi v_s)^{1/2}$. This distance is shown in Fig. 5a as a function of the mass eruption rate, for the cases in which we assume 0.25 (lower curve), 0.5 and 0.75 (upper curve) of the fluid in the fountain separates from the flow and rises upwards. It is seen that this leads to dispersal distances of 1–5 km for the mass eruption rates shown in the Fig. 5a.

We also note that typical ambient current speeds in the deep sea are of order $0.01 \,\hbox {ms}^{-1}$, while our model predicts that the typical height of the intrusions above the seafloor are of order 300–500 m. Given particle fall speeds of order 0.1–$0.4\, \hbox {ms}^{-1}$, the settling time will then be of order 1000 s. In this time, the lateral transport by the ambient currents is relatively small compared to the initial radial dispersal of the pyroclasts by the spreading intrusion.

The remainder of the erupting material in the fountain is expected to collapse on the seafloor leading to formation of a submarine pyroclastic suspension flow^8,50–52^. These gravity currents have a complex structure that may become vertically stratified by turbulent mixing on the top surface and particle sedimentation at the base^46,48,53–56^. Furthermore, once most of the particles have settled from this flow, the residual fluid-particle mixture may undergo a buoyancy reversal and lift off from the seafloor, as shown in Fig. 2a, which may result in a reduced run-out distance^48,51,52,57^. Noting these complexities, it is valuable to develop a simple estimate for the run-out distance of such currents. To this end we have adopted an idealised model in which we assume the flow remains well mixed and in which the particles sediment from the flow, of volume flux $V_w$, as it spreads radially^46,58^. This leads to the evolution of the particle concentration in the current, *C*(*r*, *t*) given by

9 $$\begin{aligned} V_w \frac{dC}{dr}= -\frac{v_s C}{h} \end{aligned}$$

so that

10 $$\begin{aligned} C(r)= C(0) e^{- (\pi v_s r^2)/ V_w} \end{aligned}$$

Here $V_w$ is the volume flux in the collapsing fountain and *h* is the thickness of the pyroclastic flow. Using our estimates for the volume flux in the fountain, as calculated from our near-source fountain model, in Fig. 5c we present an estimate for the length scale *R*, over which the concentration of particles, *C*, and hence the deposition rate, has decreased by a factor 1/*e* of the initial value in the pyroclastic flow

11 $$\begin{aligned} R=\sqrt{V_w/(\pi v_s)} \end{aligned}$$

Using this relation, we illustrate that small pyroclasts may be dispersed up to several km’s from the source vent in a dense flow (Fig. 5c). It is important to note that this estimate is calculated assuming that the surrounding topography is completely flat, however variations in the local topography may modify the run-out distance of the flow. It would be of interest to carryout further detailed experiments to explore the effect of variations on topography on the run-out distance of the flow^8,50,59–61^.

## Discussion

There are numerous technical and logistical challenges associated with accessing deep-submarine volcanic deposits, and it is difficult to obtain spatially comprehensive data sets about the deposits. However it is of interest to compare the present model with the available data to help constrain estimates of the mass eruption rates of particular eruptions. The submarine eruption of the Havre volcano, New Zealand in 2012 provided the opportunity for extensive in situ sampling and the characterisation of seafloor clastic deposits^14–16^. Although the process of fragmentation and the eruption sequence are complicated, Murch et al^14,15^ suggest that the generation of the extensive ash with lapilli unit found at the Havre volcano, occured in two phases. The authors infer that the far reaching ash deposits, Subunits 1 and 2, were produced contemporaneously with the giant pumice deposit and the voluminous pumice raft. We expect that the presence of large pumice blocks, which may or may not be positively buoyant, would have a significant effect on the dynamics of the eruption column, which are not included in our model. However, of particular interest to this study are the products of a second stage of pyroclastic activity and the identification of a single correlated ash layer (Subunit 3) over an area $>10$ $\hbox {km}^2$, that displays both thining and fining with distance from the inferred source. Murch et al^14^ conclude that this unit was produced from venting of primarily fine grained pumice, and so it is of interest to compare this phase of the eruption with our model. By assuming ambient conditions similar to those found at the Havre volcano^14,15^ and estimating the fall speed of the pyroclasts as a function of grain size^15,62^, the three circles in Fig. 5b show the range of dispersal distances of samples taken from Subunit 3^15^ with approximate fall speeds of 0.01, 0.025 and 0.1 $\hbox {ms}^{-1}$. This suggests that this explosive stage of the eruption was characterised by a minimum eruption rate of at least order $10^7$ $\hbox {kg s}^{-1}$.

**Figure 6:**
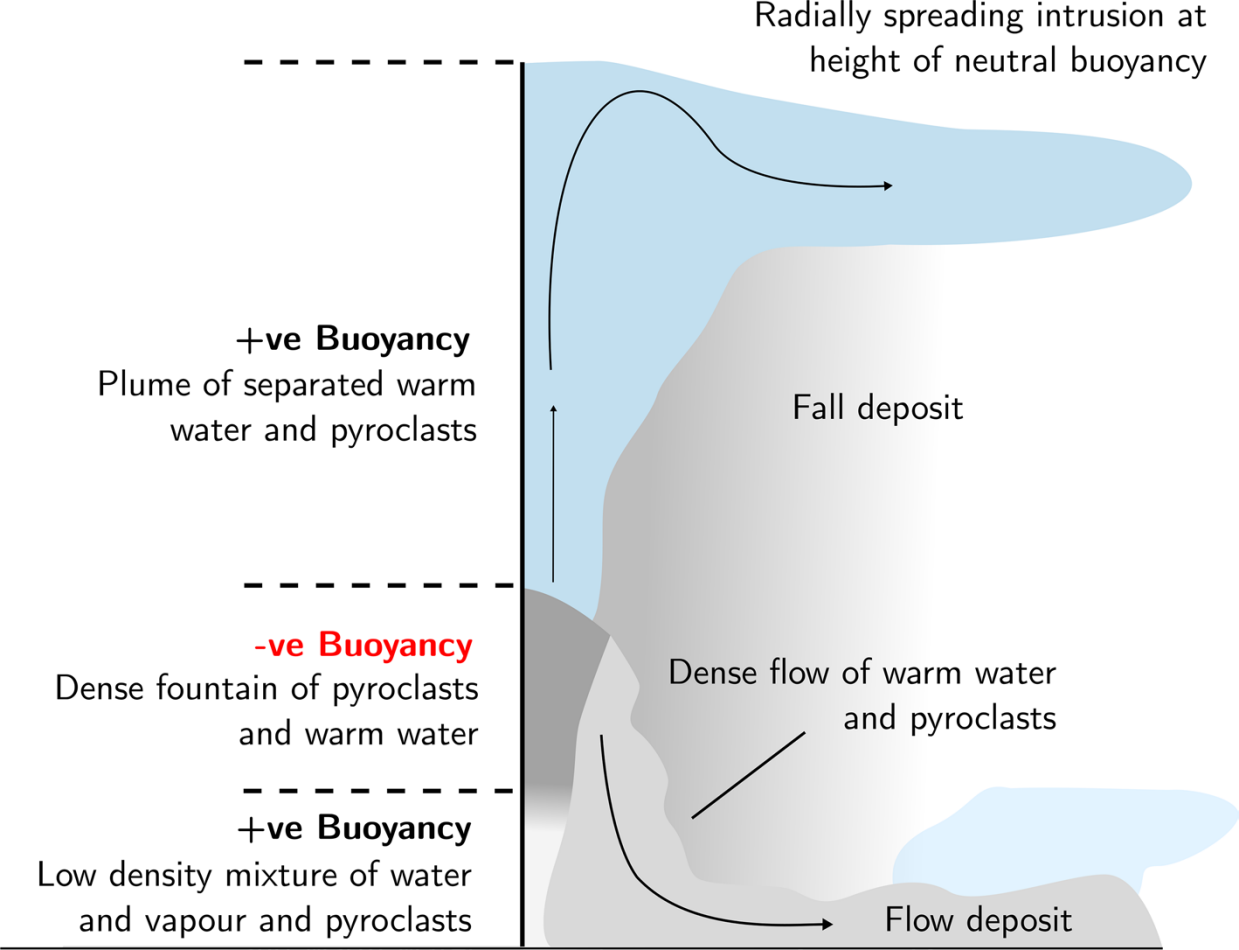
Schematic diagram illustrating the dynamics of deep-submarine explosive eruptions.

The data from Gakkel ridge is somewhat more sparse, and there is some uncertainty as to the location of the source vents; nonetheless, abrupt changes in the volcaniclastic deposit thicknesses are inferred to represent contacts between units of different ages, and from observations of the near-seafloor, these distinct deposits are seen to cover areas of up to $2 \,\hbox {km}^2$^2,12,22^. If we consider the median blocky clast from the sampled deposits ($d\sim 1\,\hbox {mm}$, $v_s\sim 0.1\,\hbox {ms}^{-1}$)^2^, our model suggests that for the dispersal of pyroclasts over distances of 1–1.5 km, the mass eruption rate would be of order $10^6\,\hbox {kg s}^{-1}$ (Fig. 5b).

The processes at play in a deep-submarine explosive eruption are quite different from a sub-aerial eruption and involve the complex interplay of sedimenting particles, the entrainment, heating and perhaps vaporisation of the seawater near the vent, and the density stratification of the ambient water column (Fig. 6). After an initial zone of mixing, in which the water-pyroclast mixture becomes very buoyant, the continued mixing of water leads to a reversal in the buoyancy, and a dense turbulent jet of particles and warm water forms. A buoyant plume can rise from this fountain following the partial sedimentation of the particles, allowing the warm water and residual particle load to rise from the fountain, while the remainder of the fountain collapses to form a radially spreading submarine pyroclastic flow. This new dynamic picture of submarine eruption processes provides some of the building blocks to help interpret submarine explosive eruptions and their deposits.

## Methods

### Experimental methods

 The analogue experiments presented in this paper were carried out in a Perspex tank with an internal cross-section of 50 cm $\times$ 50 cm, filled with an aqueous saline solution to a depth of 40 cm. The double bucket method^63^ was used to obtain a linear density stratification for some of the experiments. The density of the ambient and injected fluid was measured using a refractometer (Atago Palette PR-32 $\alpha$ digital refractometer, accuracy of $\pm 0.1$). The variation in the temperature of the ambient and injected fluid was determined to be less than 0.5 $^{\circ }\hbox {C}$ in each experiment. The density contrast associated with this temperature range is of order $10^{-4}$ whereas the variation in the density associated with changing either the salt concentration or the particle mass fraction of the fluid is order $10^{-2}$. Therefore we conclude the variation in temperature in our experiments does not have a significant impact on our density measurements.

**Figure 7:**
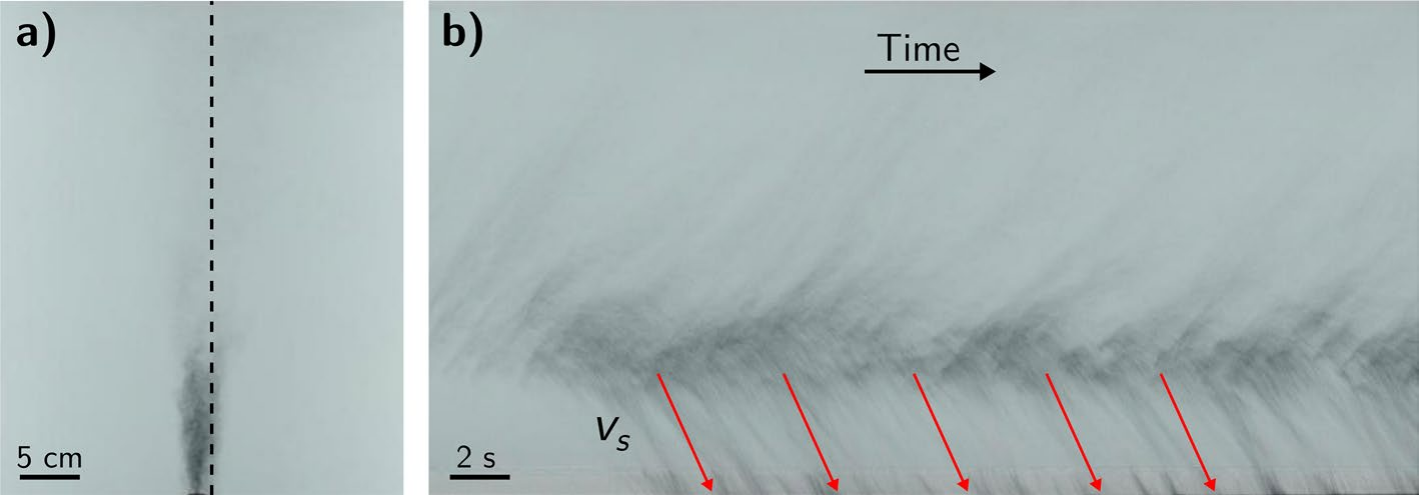
Images from experiment 1 used to measure fall speed of particles. (**a**) Snapshot of experiment 20 s after start of injection. Black dashed-line indicates location of vertical time-series. (**b**) Time-series taken at the edge of particle laden zone showing descending pulses of particles. The red lines indicate the stokes settling speed, $v_s$, of the particles.

To generate the particle-laden fountains, we placed a mixture of particles and source fluid in a beaker and used a mechanical stirrer to continuously mix the particles and fluid. This mixture was then supplied to the experimental tank using a Watson Marlow peristaltic pump at a constant volume flux. The source nozzle in the tank had a diameter of 5 mm and was located at the bottom of the tank. The tank was back lit using an electronic light sheet (W&Co) and each experiment was recorded using a Nikon D5300 digital camera with a frame rate of 50 Hz. The fall speed of the particles was estimated using the equation

12 $$\begin{aligned} v_s = \frac{2}{9} \frac{\rho _p - \rho _w}{\mu _w} g \left( \frac{D_p}{2}\right) ^2 \end{aligned}$$

where $\rho _p = 3210 \,\hbox {kgm}^{-3}$ is the density of the particles, $\rho _w$ is the density of water, $\mu _w$ is the dynamic viscosity of water and $D_p$ is the average diameter of the particles. For experiments 1–3 the particles separate from the fountain fluid as the fountain rises and the particles sediment to the tank floor^32,45^. To measure the settling speed of the particles in these experiments we created a time series of a vertical line at the edge of the particle-laden zone (Fig. 7). These time series reveal a series of descending clouds of particles below the top height of the fountain. By measuring the gradient of the streak-lines we are able to determine the speed of the descending clouds. This speed is within 5% of the Stokes settling speed (Eq. 12).

To determine the relative flux of particles rising in the buoyant plume and collapsing to the tank floor ($Q_{p\ (plume)}/ Q_{p\ (source)}$), experiments 2–22 were repeated without dying the interstitial fountain fluid. The images captured during each experiment were analysed using MATLAB. By removing the background from each image, we measured the colour intensity of the area above the fountain over time to estimate the concentration of particles rising in the buoyant plume. We compared these measurements to measurements of colour intensity taken at the height of the nozzle from a set of reference experiments in which all the particles collapsed to the tank floor.
